# Evaluating content validity for a culturally relevant wellness scale for women: the HER-WELL scale

**DOI:** 10.3389/fgwh.2026.1749005

**Published:** 2026-05-01

**Authors:** Heba Yaagoub AlNujaidi, Saja A. Al-Rayes, Arwa Alumran, Danya Y. AlNujaidi, Ibrahim A. Alhafid, Ghada F. Al Yousif

**Affiliations:** 1 College of Public Health, Imam Abdulrahman Bin Faisal University, Dammam, Saudi Arabia; 2 College of Medicine, Imam Abdulrahman Bin Faisal University, Dammam, Saudi Arabia; 3 Department of Family and Community Medicine, College of Medicine, Imam Abdulrahman Bin Faisal University, Dammam, Saudi Arabia

**Keywords:** content validity, cultural adaptation, instrument validation, psychometric development, women's wellness, HER-WELL scale

## Abstract

**Introduction:**

Wellness encompasses multiple dimensions that vary across populations, particularly among Saudi women, whose experiences are shaped by cultural, social, and religious factors. These factors shape the way wellness is perceived, pursued, and preserved. Despite growing attention to women's health, existing wellness scales often lack cultural relevance and gender sensitivity. In response, this study centers on assessing face and content validity as an initial validation step toward developing a culturally relevant wellness scale specifically for Saudi women, grounded in empirical evidence and rooted in their lived experiences.

**Methods:**

The wellness concepts and item pool were developed from a previous qualitative phase and a systematic review, which together identified eight core wellness dimensions. Face and content validity were assessed using a mixed-methods approach that combined qualitative and quantitative techniques.

**Results:**

A panel of multidisciplinary experts reviewed the initial pool of 71 items for clarity, relevance, and necessity. Based on their feedback, the scale was refined to 60 items covering the eight core wellness dimensions. These content-validity results provide the conceptual and cultural foundation for the instrument.

**Discussion:**

These content-validity results provide the conceptual and cultural foundation for the instrument and serve as a necessary starting point for future psychometric testing (e.g., CFA, PLS-SEM). Moreover, the mixed-methods validation approach used in this study provides a practical model for future researchers seeking to develop and validate measurement tools.

## 1 Introduction

Imagine waking up each day feeling physically healthy but emotionally drained, socially disconnected, and intellectually unstimulated. This is a reality for many despite having access to excellent healthcare services. In our daily lives, we often focus on health, which is typically controlled and evaluated by the resources and regulations of institutional systems. Health initiatives, policies, and services are designed to maintain and improve physical health, emphasizing medical care and disease prevention. Hippocrates, the father of medicine, stated that

“Everyone has a doctor in him or her; we just have to help it in its work. The natural healing force within each of us is the greatest force in getting well. Our food should be our medicine. Our medicine should be our food.” (1).

This quote highlights the concept of wellness, which goes beyond the absence of illness to encompass a proactive approach to health. Wellness is a broader concept that extends beyond physical health. It is a dynamic process of change and growth, determined by the individual. Unlike health, which is often externally managed, wellness is inherently personal and self-directed (2, 3). While health can be measured by tangible metrics such as blood pressure and cholesterol levels, wellness encompasses multiple dimensions, including emotional, social, intellectual, spiritual, and physical. It reflects a holistic balance that one strives to achieve through conscious choices and lifestyle changes (4–6). Wellness is also distinct from eudaimonic well-being, which emphasizes living a life characterized by purpose, meaning, and self-actualization, often an outcome (7). In contrast, wellness generally denotes the proactive and holistic maintenance of health across multiple dimensions, including mental, physical, and social aspects (8).

However, achieving wellness is not a uniform journey for everyone. Cultural, social, and religious contexts significantly influence how individuals pursue and experience wellness (9–12). This is particularly true for Saudi women, who navigate unique cultural landscapes that shape their wellness experiences. According to recent global analysis, there is a gender imbalance in well-being, revealing that women tend to have more negative affect and less positive affect, while expressing life satisfaction that is similar to or exceeds that of men (13). This paradox highlights the limitations of wellness measures in capturing the full scope of women's lived experiences. Existing wellness scales have been developed for general populations and often lack both gender sensitivity and cultural alignment, making them insufficient for accurately assessing women's wellness. It also underscores the need for culturally sensitive and gender-specific measurement tools that accurately reflect women's wellness.

A recent systematic review conducted in the previous phase by the researchers found that none of the existing wellness theories or models were specifically developed for women or explicitly addressed the gender gap in wellness (14). Even among studies explicitly focused on scale development, women-specific needs and culturally grounded domains were largely overlooked (15–24). This gap underscores the need for a wellness instrument that is both gender-sensitive and culturally relevant for Saudi women.

Building on a prior qualitative study and systematic review, the authors developed an item pool representing eight core dimensions of women's wellness as conceptualized by Saudi women (14). In line with best-practice frameworks for scale development, the next essential step is to establish robust evidence of face and content validity before proceeding to construct validity and reliability testing (25, 26). Face validity examines whether items appear clear and appropriate to intended respondents and experts, whereas content validity evaluates the extent to which items adequately represent the construct domain (27, 28).

The present study, therefore, aims to evaluate the face and content validity of a newly developed, culturally relevant Women's Wellness Scale for Saudi women using a mixed-methods expert review approach. This study reports only the initial face and content validity phase of HER-WELL development; construct validity, reliability, and criterion-related validity will be addressed in subsequent studies, consistent with staged scale-development frameworks (25). This study represents the first phase of the HER-WELL Scale development, focusing on establishing content validity. Further psychometric validation, including construct validity and reliability testing, is required before the scale can be recommended for full clinical or research use.

## 2 Methods

### 2.1 Instrument: HER-WELL scale

This study represents the initial content validation phase of HER-WELL Scale development, employing an expert-judgment content validity approach. The HER-WELL (Holistic Empowerment and Resilience-Wellness) Scale was developed by the authors to assess multidimensional wellness among Saudi women. It is grounded in the Eight Dimensions of Wellness framework (29), which was theoretically refined and culturally adapted based on a prior systematic review and qualitative research with Saudi women to ensure relevance to their lived experiences.

### 2.2 Scale development and validation

**Figure 1:**
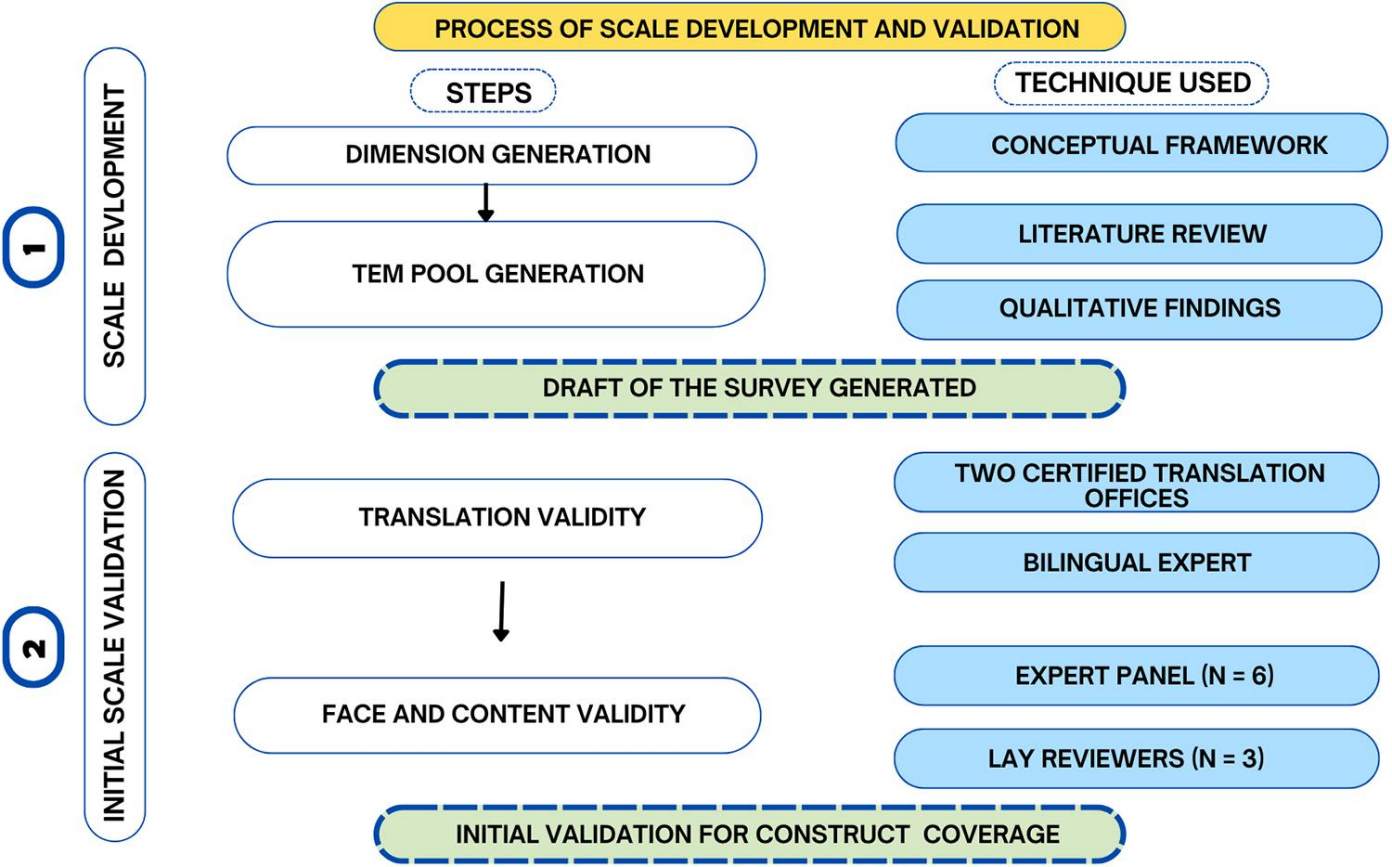
Process of scale development and initial validation for the Women's Wellness Scale.

This research adopted with minor modifications, the three-phase strategy outlined by Boateng et al. (25) for developing and validating scales for health, social, and behavioral research. This farmwork was recommended Elangovan and Sundaravel (26), as it is a widely cited step-by-step guide outlining best practices for developing and validating health, social, and behavioral research scales. The first phase consists of item development, including identifying the dimensions and generating items. The authors then assessed the scale's translation validity, followed by face and content validity. Phase two focuses on pre-testing, sampling, scale administration, item reduction, and extraction of latent constructs. Phase three assesses dimensionality, reliability, and further forms of validity (e.g., construct, criterion). In the present paper, we report only the phase-one work on item development and the expert-based assessment of face and content validity, which form the foundation for subsequent psychometric testing. See Figure 1.

Best-practice guidelines in scale development highlight the importance of transparent item generation and documentation of item sources (26). Items can be generated via deductive strategies, drawing on existing theory, literature, and validated instruments, or via inductive strategies, grounded in qualitative data such as interviews, focus groups, and observations (30, 31). It is essential to inform expert reviewers how each item was created, even when items are adopted without alterations; they must be validated for cultural relevance, and their sources should be clearly documented (26). Regardless of origin, items must be reviewed for cultural relevance and clearly linked to the construct definition to support later validity assessment (32). In addition, cross-cultural instrument guidelines emphasize rigorous translation and adaptation procedures to ensure semantic and conceptual equivalence across languages (33, 34).

#### 2.2.1 Identifying the domains and item generation of the scale

Domains and items were identified using (a) a prior systematic review of wellness theory and scales and (b) a qualitative study exploring how Saudi women conceptualize wellness (14). During scale development, cultural adaptation was carefully considered by incorporating findings from the previous qualitative research phase and by modifying a previously developed Western wellness inventory based on an eight-dimensional framework (35). In this study, only conceptually relevant aspects were adapted, and culturally modified items were developed based on the qualitative findings to align with Saudi women's experiences. The adaptation focused on preserving conceptual meaning while modifying cultural expressions, norms, and examples. This approach ensured that the scale items were not only linguistically accurate but also culturally relevant and meaningful for Saudi women (36).

The final conceptual model included eight wellness dimensions that served as primary domains for measuring overall wellness. Items were generated using a multi-source approach (37). Included: (1) qualitative findings from interviews conducted in the previous phase, (2) Swarbrick's theoretical wellness frameworks, (3) adapted items from the Western Wellness Inventory (35), and (4) new items generated from essential factors that contribute to women's wellness in the literature review (38–44)

#### 2.2.2 Translation validity

Arabic and English are the main languages used in Saudi Arabia; therefore, the scale was developed in both languages. Translation and cross-cultural adaptation followed recommended guidelines for measurement instruments (34, 36, 45). Forward translation of the original English instrument into Arabic was conducted independently by an authorized translation office (Shumookh AlNajah). Back-translation from Arabic to English was then performed by a second certified translation office (Meaning Company). A bilingual expert familiar with Saudi culture reviewed both versions and reconciled discrepancies to ensure semantic and conceptual equivalence between the original and translated versions. The harmonized Arabic and English versions were used for the subsequent face and content validity assessment.

#### 2.2.3 Face and content validity

Face and content validity were evaluated using expert review, in line with established guidelines (46, 47). Face validity and content validity are two distinct concepts that are sometimes conflated (47). In simple terms, face validity occurs when the items are clear and easy for participants to read (48). Content validity refers to the extent to which the items are relevant to measuring the construct they are intended to assess (46). Based on the literature review, three criteria were chosen to reflect face and content validity: clarity, relevancy, and essentiality (49–51).

Each item was rated on a four-point Likert scale: 1 = Not clear/relevant/essential; 2 = Needs major revision; 3 = Clear/relevant/essential but requires minor revision; 4 = Very clear/relevant/essential. Experts individually rated each item for clarity, relevance, and essentiality. The three criteria were assessed separately and not combined. Discrepancies between ratings were reviewed during qualitative feedback, and items with low scores in any criterion were marked for revision.

To gather richer input, open-ended comments were collected to gather qualitative feedback, enabling experts to offer specific suggestions for improving clarity, relevance, and comprehensiveness. A dedicated comment section was included for each item to allow detailed input. After the review, an open-ended question invited panelists to share overall feedback: “Do you have additional comments or recommendations for improving the instrument?” This process ensured that both quantitative ratings and qualitative insights informed item refinement.

Following the guideline used, we begin with creating the scale blueprint, which represents the operational definition of the primary construct and its dimensions, as shown in Table 1. Face and content validity were conducted using cognitive interviews. Cognitive interviewing is a technique for obtaining expert feedback on item format and content through verbal probing and think-aloud techniques (52–54). During cognitive interviews, experts were asked probing questions such as: “What does this item mean to you?”, “Is anything confusing or culturally misaligned?”, and “How would you rephrase this item?” Responses were recorded and categorized into clarity issues, cultural adaptation needs, or content redundancy. These qualitative categories were triangulated with quantitative indices (I-CVI, Aiken's V, Kendall's W) and open-ended written comments to guide item revision.

**Table 1:** Scale blueprint (operational definition).

Definition of Wellness	Wellness is the active pursuit of activities, choices, and lifestyles that lead to a state of holistic health.	8 Diminsions with a Total Questions 65
Physical Wellness	Physical wellness involves maintaining a healthy body through exercise, balanced nutrition, and sufficient sleep. It also includes the ability to manage physical health effectively, including accessing healthcare and monitoring personal fitness. This dimension excludes emotional or mental health aspects, focusing solely on the physical state.‏	11 Items
Emotional Wellness	Emotional wellness refers to the ability to manage personal struggles and successes, maintain healthy family relationships, and feel secure and safe.	9 Items
Home Wellness	Home wellness pertains to the physical and emotional environment of the home. It includes creating a space that supports relaxation, safety, and well-being inside and surrounding home. This dimension does not extend to external environments like workplaces or public spaces.	6 Items
Social Wellness	Social wellness is the ability to build and sustain meaningful relationships with family, friends, and the community. It includes the impact of cultural strengths and constraints, as well as the role of nuclear and extended family.	9 Items
Spiritual Wellness	Spiritual wellness reflects an individual's connection to their faith and values, encompassing practices like prayer, worship, and meditative reflection (*Tafakkur*). It includes trust and attachment to God, respect for parental obedience, and engaging in acts of charity. This dimension is not about religious formalities alone but how faith provides inner peace, purpose, and guidance in life	9 Items
Financial Wellness	Financial wellness involves achieving financial stability and independence, enabling individuals to meet their needs and plan for the future without undue stress.	6 Items
Mental wellness	Mental wellness refers to the ability to manage mental burdens, cope with stress, and maintain psychological resilience.	9 Items
Occupational Wellness	Occupational wellness encompasses job satisfaction, a healthy work environment, and balancing family-centered responsibilities with personal growth and lifelong learning.whether paid or unpaid.	6 Items emplyed
		6 Items Unemplyed

#### 2.2.4 Participants (expert panel)

The main participants in the face and content validity phase were members of an expert panel, comprising content experts and lay experts (55). The research team purposively recruited content experts through diverse professional networks and community channels to ensure disciplinary and experiential diversity. Content experts were selected purposively to reflect both theoretical expertise in the construct being measured and practical familiarity with its intended clinical application. Specifically, experts were drawn from psychology, psychiatry, women's health, and general medicine — the disciplines most likely to administer and interpret the HER-WELL Scale in real-world settings. Content experts were professionals with research or clinical experience in women's health, psychology, psychiatry, or psychometric scale development, each with at least five years of relevant experience. By selecting experts who embodied both roles simultaneously, the review process ensured that items were evaluated not only for theoretical accuracy but also for clinical relevance and practical interpretability across the disciplines in which the scale is intended to be used.

Lay experts were Saudi women purposively selected from participants who had taken part in the previous qualitative phase of the study, ensuring that the instrument reflected authentic lived experiences of the target population. Lay experts contributed a user-centered perspective by assessing whether the items were understandable, relevant, and culturally appropriate from the viewpoint of the intended respondents. No exclusion criteria were applied beyond these inclusion requirements.

To ensure a structured and consistent evaluation process among experts from different disciplines, all panel members received standardized instructions, provided both verbally during the cognitive interview and in writing prior to the review. Including a brief introduction to the study, an explanation of the wellness construct and its eight dimensions, and detailed guidance on using the 4-point Likert scales for clarity, relevance, and essentiality. Experts were also provided with space for item-level comments and open-ended feedback to capture qualitative suggestions. Convergence across expert perspectives was achieved through iterative review rounds and the research team's synthesis of feedback. The process continued until no new suggestions or themes emerged, indicating data saturation (see Table 1).

The final panel size was determined in line with established recommendations. Rubio et al. (47) Suggest an optimal range of five to ten experts. While, Roebianto et al. (46) note that the proportion of experts for content validation varies across the literature. This variation is influenced by research parameters, instrument types, and the availability of experts. Engaging at least three experts from both content and lay categories is advisable to ensure a range of perspectives. Review rounds continued until no new suggestions or themes emerged, indicating data saturation (56). This iterative process lasted approximately two weeks, at which point the panel was considered complete. The final expert panel consisted of nine members: six content experts — two women's health specialists, one psychologist, one psychiatrist, and two psychometric development experts — and three lay experts representing the target population.

Investigator triangulation was partially achieved by including experts from diverse disciplines and backgrounds, thereby contributing varied perspectives on item clarity, relevance, and cultural appropriateness (57). Convergence across expert perspectives was achieved through iterative review rounds, triangulation of quantitative ratings with qualitative feedback, and synthesis by the research team.

### 2.3 Data analysis

This study employed methodological triangulation to enhance the rigor of the face and content validity assessments, integrating quantitative and qualitative approaches to ensure a comprehensive evaluation (58–60). Quantitative data were analyzed using established content validity indices (I-FVI, I-CVI, Aiken's V, S-CVI, Kendall's W) to evaluate expert agreement on item clarity, relevance, and essentiality. In parallel, qualitative feedback gathered through cognitive interviews and open-ended reviewer comments was analyzed using structured content categorization to identify issues related to linguistic clarity, cultural sensitivity, and content redundancy (54, 61). Cognitive interviewing is a structured method in which participants articulate their thought processes as they respond to scale items, employing either think-aloud protocols or verbal probing (54). These categories were then triangulated with quantitative indices (I-CVI, Aiken's V, Kendall's W) to guide item revision decisions.

Integrating both data types allowed for a comprehensive and nuanced refinement of the scale, aligning with recommendations for mixed-methods validation (57, 59, 60). Triangulation occurred by comparing (1) quantitative ratings, (2) qualitative cognitive interview feedback, and (3) written comments from experts. Items flagged across multiple data sources were prioritized for revision. This strengthened validity by ensuring no single method dominated the evaluation.

Data analysis was conducted using Microsoft Excel for the entry and systematic tabulation of content validity indices, with all expert codes and comments recorded accordingly. SPSS was used to calculate Kendall's Coefficient of Concordance. Face validity was calculated using four measures: I-FVI (item-level face validity index), S-FVI/Ave (scale-level face validity index based on the average method), and S-FVI/UA (scale-level face validity index based on the universal agreement method) (28). See Table 2. Content validity was calculated using five measures: I-CVI (Item-Level Content Validity Index), S-CVI (Scale-Level Content Validity Index) by S-CVI/Ave and S-CVI/UA, Aiken's V, and Kendall's Coefficient of Concordance (W) (51, 63, 64). See Table 3.

**Table 2:** Face validity measurements.

Face Validity Measurements	Definition	Formula	Interpretation Reference
I-FVI (item-level face validity index)	The proportion of rater giving an item a clarity and comprehension rating of 3 or 4.	$I-\mathrm{FVI}=\frac{\begin{array}{c} \mathrm{Number}\;\;\mathrm{of}\;\mathrm{experts}\;\mathrm{rating}\;\mathrm{the}\;\mathrm{item}\;\\ \mathrm{as}\;\mathrm{relevant}\;(3\;\mathrm{or}\;4) \end{array}}{\mathrm{Total}\;\mathrm{Number}\;\mathrm{of}\;\mathrm{Experts}}$	A minimum I-FVI of.78 for 6 to 10 experts according (28, 51, 62)
			According to (27): > 0.79 (appropriate); 0.70– 0.79 (need revision); < 0.70 (eliminated).
S-FVI/Ave (scale-level face validity index based on the average method)	The average of the I-FVI values for all items in the scale.	$S-\mathrm{FVI}/\mathrm{Ave}=\frac{\sum I-\mathrm{FVI}}{\mathrm{Total}\;\mathrm{Number}\;\mathrm{of}\;\mathrm{Items}}$	According to (28)
			S-FVI/Ave ≥ 0.80: Good face validity.
			S-FVI/Ave < 0.80: The scale may require revisions
S-FVI/UA (Scale-level face validity index based on the universal agreement method	The proportion of items that achieved universal agreement among experts (i.e., all experts rated the item as clear)	$S-\mathrm{FVI}/\mathrm{UA}=\frac{\begin{array}{c} \mathrm{Number}\;\mathrm{of}\;\mathrm{items}\;\mathrm{with}\;\\ \mathrm{universal}\;\mathrm{agreement} \end{array}}{\mathrm{Total}\;\mathrm{Number}\;\mathrm{of}\;\mathrm{Items}}$	According to (28)
			S-FVI/UA ≥ 0.80: Strong universal agreement.
			S-FVI/UA < 0.80: Some items may need revision
Aiken's V^a^	Is a statistical formula designed to assess the validity of test items	$V=\frac{\sum r-l0}{n-(c-1)}$	According to (46)
			V close to 1 = High agreement among experts (strong validity)
			V close to 0 = Low agreement (weak validity)

a V, Aiken's validity coefficient (ranges from 0 to 1); **∑(r−l0)**, Sum of each rater's score minus the lowest possible score; **r**, Rating given by each expert; **l_0_**, Lowest possible score on the rating scale; *n*, Total number of raters (experts); **c**, Number of categories in the rating scale (e.g., a 4-point Likert scale has four categories).

**Table 3:** Content validity measurements.

Content Validity Measurements	Definition	Formula	Interpretation Reference
I-CVI (Item-Level Content Validity Index)	The proportion of rater giving an item a relevance rating of 3 or 4.	$I-\mathrm{CVI}=\frac{\begin{array}{c} \mathrm{Number}\;\mathrm{of}\;\mathrm{experts}\;\mathrm{rating}\;\mathrm{the}\;\mathrm{item}\;\\ \mathrm{as}\;\;\mathrm{relevant}\;(3\;\mathrm{or}\;4) \end{array}}{\mathrm{Total}\;\mathrm{Number}\;\mathrm{of}\;\mathrm{Experts}}$	A minimum I-CVI of.78 for 6 to 10 experts according (28, 51, 62)
			According to (27): > 0.79 (appropriate); 0.70– 0.79 (need revision); < 0.70 (eliminated).
S-CVI/Ave (Averaging Calculation Method)	The average of the I-CVI values for all items in the scale.	$S-\mathrm{CVI}/\mathrm{Ave}=\frac{\sum I-\mathrm{CVI}}{\mathrm{Total}\;\mathrm{Number}\;\mathrm{of}\;\mathrm{Items}}$	According to (28)
			S-CVI/Ave ≥ 0.80: Good Content validity.
			S-CVI/Ave < 0.80: The scale may require revisions
S-CVI/UA (Universal Agreement Calculation Method)	The proportion of items that achieved universal agreement among experts (i.e., all experts rated the item as clear)	$S-\mathrm{CVI}/\mathrm{UA}=\frac{\begin{array}{c} \mathrm{Number}\;\mathrm{of}\;\mathrm{items}\;\;\mathrm{ith}\;\\ \mathrm{universal}\;\mathrm{agreement} \end{array}}{\mathrm{Total}\;\mathrm{Number}\;\mathrm{of}\;\mathrm{Items}}$	According to (28)
			S-CVI/UA ≥ 0.80: Strong universal agreement.
			S-CVI/UA < 0.80: Some items may need revision
Aiken's V^a^	Is a statistical formula designed to assess the validity of test items	$V=\frac{\sum r-l0}{n-(c-1)}$	According to (46)
			V close to 1 = High agreement among experts (strong validity)
			V close to 0 = Low agreement (weak validity)
Kendall's Coefficient of Concordance (W)	It measures the agreement among multiple raters ranking a set of items, focusing on consistency rather than chance-based agreement.	$W=\frac{12\sum R_{i}^{2}-3N^{2}{\left(m+1\right)}^{2}}{m^{2}(N^{3}-N)}$	According to (65)
			W = 1: Perfect agreement among all raters.
			W = 0: No agreement, rankings are completely random

a **V**, Aiken's validity coefficient (ranges from 0 to 1); **∑(r−l0)**, Sum of each rater's score minus the lowest possible score; **r**, Rating given by each expert; **l_0_**, Lowest possible score on the rating scale; *n*, Total number of raters (experts); **c**, Number of categories in the rating scale (e.g., a 4-point Likert scale has four categories).

The interpretation of each measure is as follows: the item validity index ranges from 0, indicating no agreement, to 1, indicating high agreement (46).. Aiken's V ranges from 0 to 1: V close to 1 = High agreement among experts (strong validity), and V close to 0 = Low agreement (weak validity) (46). Kendall's Coefficient of Concordance (W) ranges from 0, indicating no agreement with completely random rankings, to 1, indicating perfect agreement among all raters (63, 65).

## 3 Results

To assess the face and content validity of the Women's Wellness Scale. The researchers employed nine experts from different specialties using the cognitive interview (think-aloud Technique) to assess face and content validity for both the Arabic and English versions: two women's health specialists, one psychologist, one psychiatrist, two psychometric development experts, and three lay experts. Content experts were selected based on a minimum of five years of professional or research experience in at least one of the following fields: women's health, psychology, psychiatry, or psychometric scale development. Lay experts were Saudi women representing the intended target population, selected to ensure the tool reflected authentic lived experiences across varied socioeconomic and age backgrounds. This multidisciplinary selection ensured comprehensive evaluation across both technical validity and cultural appropriateness.

### 3.1 Face and content validity assessment

#### 3.1.1 Item-Level evaluation for scale refinement

The face and content validity of the Culturally Relevant Wellness Scale for Women was evaluated to refine it and ensure its clarity, relevance, and essentiality. Face validity focused on clarity**,** while content validity assessed relevance and essentiality**.** The experts evaluated all 71 questions and answers the scale using a 4-point Likert scale (**1** = Not clear/relevant/essential, **2** = Needs major revision, **3** = Clear/relevant/essential but requires minor revision, **4** = Very clear/relevant/essential). Also, they provide comments and suggestions for revisions where necessary. The answers were evaluated based on the expert review comments, CVI, Kendall's W, and Aiken's V. The goal was to refine the items based on expert consensus and determine which items should be retained, revised, or removed. See Supplementary Data Sheet 1.

Most items met the validity thresholds and were retained. These were the items that met the threshold for clarity, relevance, and essentiality (I-FVI ≥ 0.78, I-CVI ≥ 0.78, Aiken's V ≥ 0.75). For example, “*I exercise for at least 20 to 30 min, three times a week.”* (I-FVI = 1, Aiken's V = 0.963, I-CVI = 1). The items that need revision to enhance clarity, consistency, and structure. Refinements included linguistic adjustments (improving phrasing and grammar while maintaining meaning), structural edits (modifying sentence structure to align with the scale), and consistency updates (ensuring uniform wording across items). Items with lower expert agreement were revised for clarity or streamlined for cultural appropriateness, for example, “I actively monitor my menstrual health and seek medical advice when experiencing any irregularities or problems.” (I-CVI = 0.222, Aiken's V = 0.444, marked as revised). Twelve items were removed because experts identified them as redundant and measured the same concepts as other items. Most of these had low I-CVI, Aiken's V, and essentiality scores, indicating weak expert agreement on their relevance. For example, “*I maintain my own savings to feel financially secure and ready for future needs”* (I-CVI = 0.333, Aiken's V = 0.370) was marked for deletion due to low validity ratings. For further clarification, Table 4 provides a detailed overview of how specific items were revised based on interview feedback, outlining the modifications made to enhance clarity, relevance, and cultural appropriateness to ensure all items align with participants' experiences and perspectives. In summary, 35 items were revised before being retained, 12 items were deleted, 1 item was added, and 24 items were maintained the same. The final revised version of the scale, consisting of 60 items, integrates expert recommendations to ensure that all retained items are clear, relevant, and essential**.**

**Table 4:** Example of reviewer comments and item revisions from cognitive interview with 9 experts.

Diminsion	Item, Before	Reviwer Comments	Action taken	Item, After
Physical wellness	I manage symptoms related to hormonal changes (e.g., menstruation, pregnancy, menopause) to maintain my physical health	To mege this question with the previous question. “I actively monitor my menstrual health and seek medical advice when experiencing any irregularities or problems.”	Two question were merged	I manage symptoms related my menstrual period and hormonal changes (e.g., pregnancy, menopause) with a specialist doctor.
Emotional wellness	I express and acknowledge my feelings, whether through talking to family, engaging in hobbies, or doing acts of giving.	Recognition and aknowldging comes before expressing the feelling.	The question was revised and edited.	I acknowledge and express my feelings, whether through talking with family, pursuing hobbies, or doing acts of giving.
Enviromental wellness	I am working hard to own my own home to support my independence	This question is related more to financial aspirations like homeownership.	Deleted	**NA**
Social wellness	My friendships provide me with relaxation, positivity, and emotional balance.	Consider refining the sentence to align more closely with the intended meaning. Instead of “relaxation, positivity, and emotional balance,” a more concise and holistic phrasing	The question was revised and edited.	My friendships provide me with well-being and balance.
Spirtual wellness	Regularity in the Duha prayer brings me well-being	This is a highly specific question that may not be relevant to the overall wellness of all women.	Deleted	NA
Finicial wellness	I make informed spending decisions and avoid unnecessary financial burdens	Delete “and avoid unnecessary financial burdens”	The question was revised and edited.	I make informed spending decisions.‏
Occupational wellness	My work gives me a sense of financial independence and professional development.	Seprate it into two question since the item cover two points.	Question was seprated	My work gives me a sense of financial independence
				My work environment supports my professional development.

#### 3.1.2 Dimensions- scale level validity

Following the item-level validity assessment, the dimension-scale level analysis was conducted to evaluate the overall validity of each wellness dimension and the wellness scale.

Table 5 summarizes the scale-level and dimension-level results, including S-FVI (Face Validity), S-CVI (Content Validity for Essentiality and Relevance), and Kendall's W for inter-rater agreement. For Face validity, all dimensions showed high face validity, with S-FVI/UA and S-FVI/Ave scores nearing 1, indicating strong expert agreement on item clarity. The overall wellness scale achieved an S-FVI/UA of 0.971 and S-FVI/Ave of 0.996, confirming that most items were perceived as clear by experts and were higher than ≥ 0.80 (28).

**Table 5:** Dimension-Scale level face and content validity assessment of the wellness scale.

Dimension	No. of Items	S-FVI/UA	S-FVI/Ave	S-CVI/UA Essintiality	S-CVI/Ave Essintiality	S-CVI/UA Relavance	S-CVI/Ave Relavance	Kendall's W
Physical Wellness	10	1	1	0.727	0.899	0.818	0.919	Kendall's W = .419
Emotional Wellness	9	1	1	0.889	0.988	0.667	0.963	
Home Environment Wellness	4	0.833	0.981	0.667	0.944	0.667	0.944	
Social Wellness	7	0.888	0.987	0.667	0.951	0.667	0.951	
Spiritual Wellness	7	1	1	0.667	0.938	0.778	0.963	
Financial Wellness	5	1	1	0.500	0.852	0.833	0.889	Pvalue < .001
Mental Wellness	6	1	1	0.444	0.778	0.444	0.778	
Occupational Wellness/Employed	6	1	1	0.833	0.981	0.833	0.981	
Occupational Wellness/ Not Employed	6	1	1	1	1	0.857	0.984	
Wellness Scale	60	0.971	0.996	0.704	0.921	0.732	0.928	

For the overall content value of the scale, in terms of essentiality and relevance, the S-CVI/UA (Essentiality) for the total scale was 0.704, and S-CVI/UA (Relevance) was 0.732, which indicates that some items need revision since it is S-CVI/UA < 0.80 (28). While the S-CVI/Ave (Essentiality) reached 0.921, and S-CVI/Ave (Relevance) was 0.928, indicating Strong universal agreement on item necessity since it is above ≥ 0.80 (28). Another important test that reflects the agreement among multiple raters ranking a set of items, focusing on consistency rather than chance-based agreement, is Kendall's Coefficient of Concordance (W), which ranges between 0 and 1 (65). For this scale, W = .419, *P*-value < .001 indicates a moderate level of agreement among experts. This could be reflected by some dimensions, such as Occupational Wellness (Not Employed), which reached a perfect agreement (S-FVI and S-CVI = 1), others, like Mental Wellness (S-CVI/UA Essentiality = 0.444), showed slightly lower consensus, suggesting variability in expert opinions regarding the importance of some items.

#### 3.1.3 Overall feedback

All nine reviewers unanimously confirmed the face validity of the wellness scale across multiple criteria. These included the appropriateness of grammar, clarity, and unambiguity of items, correct spelling and sentence structure, and suitable font size and spacing. Additionally, all reviewers agreed that the scale was well-organized, culturally and linguistically appropriate for the target population, and aligned with the intended purpose and wellness dimensions. This consistent agreement across diverse experts highlights the overall clarity, coherence, and cultural relevance of the scale's presentation and content.

## 4 Discussion

The purpose of this study was to evaluate the face and content validity of a culturally relevant wellness scale for women in Saudi Arabia using expert review and cognitive interviews. This initial validation phase was essential for ensuring that the instrument reflects women's lived experiences and aligns with established conceptualizations of wellness within the Saudi context. The integration of quantitative indices and qualitative insights yielded a refined 60-item scale that shows strong early evidence of clarity, relevance, and cultural appropriateness. While wellness and well-being are theoretically distinct constructs, the former being a proactive behavioral process and the latter a psychological state (66), the present scale was developed within the wellness tradition, operationalizing eight actionable dimensions that reflect the lived experiences of Saudi women.

A key contribution of this phase was the combined use of face and content validity in a mixed-methods approach. Although these concepts are sometimes used interchangeably in the literature, face validity focuses on the surface-level clarity and interpretability of items, while content validity evaluates whether items adequately represent the intended construct domain. In this study, expert reviewers and lay participants confirmed that most items appeared clear (face validity), yet some required revision to enhance cultural alignment or semantic precision. Content validity indices further confirmed that items across the eight wellness dimensions—particularly Physical, Emotional, Social, Spiritual, and Occupational Wellness—were judged to be representative of the targeted constructs.

Quantitatively, item-level indices (I-FVI, I-CVI, Aiken's V) met recommended thresholds, indicating strong expert agreement on item clarity, relevance, and essentiality. However, some items that appeared numerically valid required modification or removal based on qualitative insights. Cognitive interview findings revealed subtle issues such as linguistic ambiguity, culturally sensitive phrasing, or conceptual overlap—issues that numerical scores alone could not capture. This reinforces the importance of mixed-methods validation, consistent with findings from cross-cultural instrument development such as the HARMONI well-being tool, which similarly benefited from integrating expert narrative feedback with psychometric indices (53).

At the dimension level, the scale showed high overall content validity (S-CVI/Ave ≥ .80 for most dimensions), demonstrating broad expert consensus. However, more variability emerged in Mental Wellness and Financial Wellness, where essentiality scores were lower (S-CVI/UA = .444 and.500). This divergence reflects the complexity of these domains for Saudi women. Mental wellness concepts—such as coping, psychological burden, or stress—are interpreted differently across clinical and cultural perspectives, leading to more disagreement among experts from varied backgrounds. Similarly, financial wellness is influenced by shifting norms around women's employment, social support systems, and financial autonomy in Saudi Arabia, which may explain difficulties in defining items deemed “essential”.

The moderate but significant Kendall's W (W = .419, *p* < .001) further indicates that expert agreement was present but not uniform, likely reflecting the panel's diverse professional backgrounds (65). Variability in expert judgments is common in early-stage scale development, especially when measuring multidimensional constructs shaped by culture, gender roles, and shifting societal expectations. This highlights the importance of continuing refinement in subsequent psychometric testing, including exploratory and confirmatory factor analysis. These discrepancies are consistent with challenges documented in other cross-cultural validation studies, where context-specific constructs often require multiple rounds of refinement before achieving consensus. The cultural context of Saudi women is central to interpreting these content validity findings. Saudi women navigate a unique intersection of Islamic values, family-centered social structures, and a rapidly evolving societal landscape shaped by Vision 2030 reforms, all of which directly influence how wellness is experienced and expressed (67). Notably, women's labor force participation has risen from from 17% in 2017 to over 36% in early 2025, reflecting shifting norms around women's employment and financial autonomy that directly shaped the variability observed in Financial and Occupational Wellness dimensions during expert review (68). Furthermore, gender norms and conservative religious beliefs have been documented as powerful influences on Saudi women's health behaviors and well-being reinforcing the need for a culturally grounded instrument rather than an adapted Western wellness scale (69, 70).

A focused literature review was conducted to identify existing women-focused scales and determine whether any provided a comprehensive, multidimensional wellness assessment. Existing measures developed specifically for women tend to be either domain-specific (e.g., mental health, spirituality, sexual or reproductive health) or stage-specific, such as instruments targeting pregnancy, postpartum recovery, menopause, or high-risk clinical groups (71–77). While these tools are psychometrically sound within their contexts, none provide a holistic assessment of wellness that integrates physical, emotional, social, spiritual, financial, mental, and occupational dimensions across the female lifespan. The present scale, therefore, provides a comprehensive, culturally grounded instrument that addresses this gap by conceptualizing women's wellness as a multidimensional, lifelong construct.

A methodological strength of this study was the use of triangulation. Combining quantitative ratings, qualitative cognitive interviews, and written expert feedback ensured that revisions were guided by multiple forms of evidence rather than numerical indices alone. Experts' diverse disciplinary backgrounds enriched the evaluation process, helping to identify items that were statistically adequate but conceptually weak or culturally misaligned. This methodological triangulation approach ensured that decisions to retain, revise, or delete items were grounded in statistical agreement and user-centered interpretation (58).

The HER-WELL Scale carries important social, clinical, and research implications. Socially, it captures wellness dimensions often overlooked in standardized measures, particularly for women in culturally specific contexts. AlNujaidi et al. (14) Noted that there is an urgent need to address the fact that current wellness frameworks have often been created without enough consideration for gender and cultural diversity. Clinically, it may support more holistic and contextually appropriate assessments of women's wellness in primary care, women's health clinics, and public health settings, enabling practitioners to identify specific wellness deficits across dimensions and tailor interventions accordingly. From a research perspective, it provides a foundation for future psychometric validation and the development of evidence-based wellness interventions targeting Saudi women, while also offering a methodological model for researchers developing culturally grounded instruments for other underrepresented populations.

Despite these strengths, several limitations warrant consideration. First, the number of lay reviewers was small, which may limit the generalizability of qualitative insights. Second, this study represents the initial content validation phase of the HER-WELL Scale development. Further psychometric analyses, including factor analysis, construct validity, and reliability testing, are necessary before full validation can be claimed. Another limitation is that only limited demographic information was gathered from experts, which limits the description of the panel's characteristics; future studies should systematically record expert demographics. Finally, Saudi Arabia is diverse, and interpretations of wellness may vary across regions, age groups, and socioeconomic backgrounds. Future testing should therefore include larger and more diverse samples to ensure broad applicability.

Overall, the findings demonstrate strong initial validity and cultural grounding for the Women's Wellness Scale. The results highlight the importance of incorporating women's lived experiences, cultural context, and input from multidisciplinary experts when developing wellness measures that are both gender-sensitive and culturally relevant.

## 5 Conclusion

This study provides strong initial evidence for the validity of a newly developed Women's Wellness Scale designed specifically for Saudi women. Through expert reviews, cognitive interviews, and quantitative indices, the scale was refined to 60 clear, relevant, and culturally meaningful items. These results suggest that the instrument captures wellness in a way that reflects women's lived experiences and aligns with the Saudi cultural context.

With face and content validation now complete, the next step is full psychometric testing, including factor analyses, reliability assessment, and measurement invariance across different groups of women. Continued validation will enable this tool to serve researchers and practitioners as a practical, culturally grounded measure to understand and improve women's wellness.

By addressing a long-standing gap in gender-sensitive and culturally relevant wellness assessment, this scale marks an essential step toward recognizing and measuring the multidimensional realities of women's well-being.

## Data Availability

The data supporting the findings of this study are available from the corresponding author upon reasonable request.

## Supplementary material

SUPPLEMENTARY DATA SHEET 1 Item level face and content validity.
